# Pharmacists’ involvements and barriers in the provision of health promotion services towards noncommunicable diseases: Community-based cross-sectional study in Northwest Ethiopia

**DOI:** 10.1186/s13690-023-01038-x

**Published:** 2023-02-25

**Authors:** Ashenafi Kibret Sendekie, Abera Dessie Dagnaw, Ephrem Mebratu Dagnew

**Affiliations:** 1 Department of Clinical Pharmacy, School of Pharmacy, College of Medicine and Health Sciences, University of Gondar, Gondar, Ethiopia; 2 Department of Pharmaceutical Chemistry, School of Pharmacy, College of Medicine and Health Sciences, University of Gondar, Gondar, Ethiopia; 3 Department of Pharmacy, College of Health Sciences, Debre Markos University, Debre Markos, Ethiopia

**Keywords:** Barriers, Community pharmacists, Health promotion, Involvement, Noncommunicable diseases, Northwest Ethiopia

## Abstract

**Background:**

Community drug retail outlets (CDROs) are among the initial healthcare facilities where pharmacists play a crucial role in preventing and managing noncommunicable diseases (NCDs). Therefore, this study assessed pharmacists’ level of involvement and barriers in the provision of health promotion for noncommunicable diseases at CDROs in Northwest Ethiopia.

**Methods:**

A community-based multicenter cross-sectional study was conducted among community pharmacists in Northwest Ethiopia from April to June 2022. Data was collected using a self-administered structured questionnaire, and analyzed using the Statistical Package for Social Science (SPSS) version 26. The level of involvement mean score difference among pharmacists was investigated using an independent samples t-test and a one-way ANOVA. Logistic regression analysis was used to examine the association between pharmacists’ level of involvement and other variables. A *p*-value < 0.05 at a 95% confidence interval (CI) was considered statistically significant.

**Results:**

A total of 285 (94.4%) participants participated in the study out of 302 approached samples. Overall, more than half (58.9%) of the participants showed a high level of involvement in health promotion. Pharmacists who had a degree and/or above (AOR = 0.03, 95% CI: 0.01–0.63; *p* < 0.001) and served a lower number of clients per day (AOR = 0.19, 95% CI: 0.04–94; *p* = 0.042) were less likely to have low involvement in health promotion services. Pharmacists who worked fewer hours per day (AOR = 3.65, 95% CI: 1.79–7.48; *p* = 0.005) were more likely to have low involvement. Lack of an appropriate area in the CDROs (52.1%) and lack of coordination with other healthcare providers (43.6%) were the most reported barriers to the provision of health promotion.

**Conclusion:**

Most pharmacists were found to have a high level of involvement in health promotion activities. A lack of an appropriate area in the CDROs and a lack of coordination with other healthcare providers were among the most reported barriers. Pharmacists might benefit from training to increase their educational backgrounds, and barriers could be addressed to enhance the pharmacist involvement.

**Supplementary Information:**

The online version contains supplementary material available at 10.1186/s13690-023-01038-x.

## Introduction

Because of social changes and unhealthy physical environments, along with unhealthy lifestyle practices, the prevalence of noncommunicable diseases (NCDs) is increasing across the globe [1]. NCDs are the major cause of global death and have been faced by both developed and underdeveloped countries. For instance, the major NCDs such as cardiovascular disease, cancer, chronic lung diseases, and diabetes account for three in five global deaths [2]. Further, according to the World Health Organization (WHO) report, it is the cause of death for 41 million people each year and accounts for 71% of all deaths globally [3]. In 2019, only cardiovascular disease-associated deaths accounted for about one-third of all deaths, and these deaths occurred prematurely in the population under 70 years old [4]. Its burden has increased, particularly in low- and middle-income countries, which account for 80% of all deaths [5, 6]. In Ethiopia, evidence from the national NCD STEPS survey reported a significantly high prevalence of raised blood pressure, hyperglycemia, dyslipidemia, and metabolic syndrome [7]. An analysis of the evidence from the Global Burden of Disease (GBD) 2016 study also reported that chronic disease contributed to 39.3% of the total death rate and 53% of the age-standardized death rate (ASDR) [8].

The occurrence of NCDs has been linked to various reasons, which are strongly related to sedentary lifestyles like smoking, obesity, physical inactivity, and an unhealthy diet, which result in NCDs such as dyslipidemia, diabetes, cardiovascular disease, renal diseases, retinopathy, and cerebrovascular diseases [9, 10]. In Ethiopia, age, alcohol intake, and abdominal obesity are also reported as significant risk factors for developing the NCDs [11, 12]. Thus, changing sedentary lifestyle behavior and appropriate management of modifiable risk factors can play a role in avoiding these risks [13]. Further, appropriate management of the modifiable risk factors has been established in both undiagnosed and known NCDs to reduce mortality and morbidity risks and improve healthcare systems [14].

Health promotion enables people to improve their health conditions and focuses on keeping people healthy [15]. Individuals and/or communities can be involved and allowed to appreciate healthy behavior and make changes focusing on the root causes and risks of developing chronic diseases [16]. The promotion may be implemented through educational and social communication activities that promote healthy conditions, lifestyles, behavior, and environments. Finally, health promotion is targeted to create and promote health literacy, good governance for health, and healthy cities. Though the practice of health promotion commonly involves the population as a whole [17], it has been implemented and is necessary for specific groups of the population, particularly in the prevention and management of chronic diseases. As a result, the National Center for Chronic Disease Prevention and Health Promotion (NCCDPHP) has promoted chronic disease prevention efforts using health promotion systems [18].

Worldwide, pharmacy practice has shifted from dispensing only to healthcare approaches, and health promotion is now considered part of current pharmacy services [19–22]. Subsequently, pharmacists are among the most frequently visited and first points of contact for the public, and they play a crucial role in preventing and managing NCDs by providing more direct interventions in medication education and disease management, resulting in improved medication adherence, achieving desired therapeutic outcomes, and improving safe medication use practices [23, 24]. Community drug retail outlets (CDROs) are the initial healthcare sites where patients could receive their medications and interventions at the community level. Community pharmacists can play a significant role in the prevention and management of chronic illnesses, cardiovascular disorders, diabetes, and metabolic syndromes, contribute to better patient care [24–29], and are considered the main contributors to the establishment of health promotion activities for individuals and communities [30]. Because patients are having difficulty accessing primary care physicians and healthcare prices are rapidly increasing, more community-based care models have been promoted.

However, pharmacists, in particular community pharmacists, have faced many challenges in providing effective health promotion activities in the public’s priorities to individuals and communities [31, 32]. Among the common barriers that affect community pharmacists in providing public healthcare are lack of knowledge and skills, confidence, and adequate training and policies; poor recognition of the healthcare system; low patient demand; and inadequate pharmacy staff [19, 29]. Lack of appropriate areas in the CDROs, increased workloads, a lack of educational material and training, and insufficient management support were the most commonly reported barriers to counseling and involvement in public healthcare activities by Ethiopian community pharmacists [21, 25]. A study on pharmacy students also showed that clients’ lack of time and interest, as well as the absence of guidelines for health promotion services, were the main barriers perceived to hinder health promotion services [33].

Evidence showed that community pharmacists play an important role in different health promotion services [19–21, 28, 34, 35]. CDROs are among the initial healthcare settings where pharmacists could play an important role in health promotion. Although some literature has investigated the role of community pharmacists in health promotion services [33], to the best of the authors’ knowledge and search, there is still limited evidence investigated to assess community pharmacists’ involvement in health promotion activities and barriers, in particular in the prevention and management of NCDs in the study areas. Therefore, this study assessed the level of involvement and barriers of community pharmacists in the health promotion activities towards the prevention and management of NCDs at CDROs in selected cities in Northwest Ethiopia.

## Methods

### Study design, settings and samples

A community-based multicenter cross-sectional study was conducted among the pharmacists working in CDROs in selected cities from April to June 2022. The participants were recruited from three cities in Northwest Ethiopia: Gondar, Bahira Dar, and Debre Tabor. The cities were selected by lottery from other cities in the Amhara regional states. The Amhara regional state was purposefully selected from other regions and administrative cities in the country. All three selected cities were considered to have comparable community pharmacy practices and practitioners. A local report revealed that there were around 195 licensed CDROs in these three cities in total as of December 2021.

Initially, considering the number of available samples in the selected cities, we calculated and found the sample size. The sample size needed to conduct the survey was calculated using the simple proportion formula: *n* = *p* (1-*p*) * (Z)^2^/d^2^; assuming a 5% margin error or degree of accuracy (d = 0.05), reliability coefficient for 95% confidence level (Z = 1.96), and *p* = 0.5 (50%) response distribution. Considering 195 active CDROs in the selected cities, we used the correction formula and added 10% contingency. The final sample size was 143 CDROs. As a result, considering the limited number of available CDROs in the selected cities, we approached all licensed community pharmacy professionals who had been working in the CDROs (more than 1 pharmacist per CDRO) during the data collection period. The survey was conducted among pharmacists who had worked at the CDROs in any selected city as qualified and licensed pharmacy professionals for at least three months. Those who were not available at the CDROs during the data collection and those who refused to participate in the study were excluded. Consequently, we initially approached 302 pharmacists, and 285 of them participated in this study.

### Operational definitions

#### Community pharmacists

In this study, this term refers to pharmacy professionals who worked at CDROs in the selected cities, regardless of their educational background.

#### Health promotion

Indicates the participation and engagement of community pharmacists in the promotion, counseling, education, and provision of services in the prevention and management of NCDs, focusing on the root causes and risks of chronic diseases.

#### The level of health promotion involvement

The degree (level) of pharmacist involvement in health promotion services. The mean health promotion involvement score was determined for each participant, with a score ranging from 1 to 5. The overall mean score of pharmacists' health promotion service involvement in NCD prevention and management was then computed out of five. The overall score was dichotomized using the mean as a cut-off point because of the lack of earlier evidence. Respondents who scored less than the overall mean score (3.82) and those who scored greater than or equal to the overall mean score had low and high levels of involvement in health promotion services, respectively.

### Data collection instruments and procedures, and data quality control methods

The data was collected using a structured questionnaire. The questionnaire was first prepared in English, then translated to Amharic (the local language), and then back to English to maintain its consistency. The questionnaire was developed after reviewing the previous literature [19–21, 25, 28, 34, 36]. The data collection instrument consisted of four parts (Supplementary file): (1) the first part consisted of socio-demographic characteristics; (2) the second part contained statements used to assess the willingness of the pharmacists to participate in the health promotion activities; (3) the third part was used to assess the level of involvement of pharmacists in promotion and counseling services in the prevention and management of NCDs; and (4) the fourth part contained different sources of barriers for community pharmacists involved in the health promotion activities.

Before the actual data collection period, the questionnaire was validated for its content by two senior clinical pharmacists and then pretested in ten CDROs (approximately 5% of the total CDROs in the selected cities). In the pretest, we administered the prepared questionnaire to the pharmacists in the ten selected CDROs and checked the questionnaire for easy understandability, clarity, and consistency. Then, using the pretest feedback, we made slight modifications to the questionnaire in terms of avoiding redundancy, making clear some ambiguous phrases, and avoiding long statements before distrusting the questionnaire. Then, the data was collected by three graduating clinical pharmacists after they received a half-day training on data collection procedures and ethical aspects. Initially, the study participants were briefed about the objectives of the study and then asked for their consent to participate in it. A self-administered questionnaire was provided to eligible participants who volunteered to participate in this study. While on data collection, the supervisor explicitly followed the data collection procedures. The data was checked for its completeness, cleanliness, and clarity every day during the data collection period.

Health promotion service items used to assess pharmacists’ health promotion involvement in prevention and management of NCDs had 14 statements with a five-point Likert scale (not at all involved, little involved, uncertain, involved, very involved) with a value of 1 up to 5 points, respectively. A reliability test for the items used to assess the health promotion involvement was performed, yielding a Cronbach’s alpha (α) value of 0.86. Factor analysis of the instrument was also performed with three factors. Factor 1 was comprised of 5 items reported on a 5-point Likert scale that explained 61.7% of the variance, with factor loadings from 0.701 to 0.821. Factor 2 also contained seven items that explained 73.4% of the variance, with factor loadings ranging from 0.591 to 0.825. The third factor also consisted of 2 items reported on a 5-point Likert scale that explained 79.3% of the variance with factor loadings from 0.744 to 0.834. The Kaiser–Meyer–Olkin (KMO) measure of sampling adequacy was also found to be adequate and had a significance level for the Bartlett's test (KMO = 0.825; *p* < 0.001).

### Data entry and statistical analysis

After the data was collected and checked for completeness and cleanliness, it was entered into EPI-info version 8. Then, it was transformed into Statistical Package for Social Science (SPSS) version 26, and after it was checked for its completeness, clarity, and consistency, it was analyzed. A histogram and Q-Q plot were used to test the normal distribution of variables. The results were presented by means and standard divisions (SD) for continuous variables and by frequencies and percentages for categorical variables. Factor analysis of the instrument was performed using the principal components method of extraction and varimax rotation. An independent samples t-test and a one-way ANOVA were employed to compare pharmacists’ mean score differences regarding their health promotion involvement in the prevention and management of NCDs. A logistic regression analysis was used to identify predictor variables in the pharmacists' involvement in health promotion. A *p*-value of < 0.05 at the 95% confidence interval (CI) was considered statistically significant.

## Results

### Sociodemographic characteristics of the study participants

Out of 302 samples approached, 285 completed the study, resulting in a 94.4% response rate. More than half (52.6%) were males, with a mean (± SD) age of 32.0 ± 8.3 years. Most of the participants (56.5%) had a lower educational background (diploma level), and a higher proportion of them (48.4%) had work experience of 1–5 years. Furthermore, most of the participants were employees (79.6%). Around two-thirds (66.3%) could not receive training related to health promotion activities (Table 1).

**Table 1:** Sociodemographic characteristics of the community pharmacists, Northwest Ethiopia (*N* = 285)

Variables		Frequency (%)	Mean (± SD)
Sex:	Male	150 (52.6)	
	Female	135(47.4)	
Average age in years:	-	-	32.0(± 8.3)
Cities where the participants worked:	Bahira Dar	116 (40.7)	
	Gondar	104 (36.5)	
	Debera Tabor	65 (22.6)	
Pharmacists’ educational level:	Druggist (diploma)	161 (56.5)	
	Bachelor's degree and above	124 (43.5)	
Work experience in years:	< 1 year	53 (18.6)	
	1–5 Years	138(48.4)	4.6(± 1.7)
	> 5 years	94 (33.0)	
Employment status:	Employee	227(79.6)	
	Owner	58(20.4)	
Monthly income (ETB):	1500–2999	86 (30.2)	
	3000–4999	126 (44.2)	4518.6(± 674.3)
	≥ 5000	73 (25.6)	
CDRO types where participants worked:	Drug store	128 (44.9)	
	Pharmacy	157 (55.1)	
Number of clients served/day:	< 50	159 (55.8)	43.5(± 17.4)
	50–100	109 (38.2)	
	> 100	17 (6.0)	
Working hours/day:	≤ 8	120(42.1)	8.6(± 2.3)
	> 8	165 (57.9)	
Training on health promotion practice:	Yes	96 (33.7)	
	No	189 (66.3)	

### Community pharmacists’ involvement in health promotion services

**Fig. 1:**
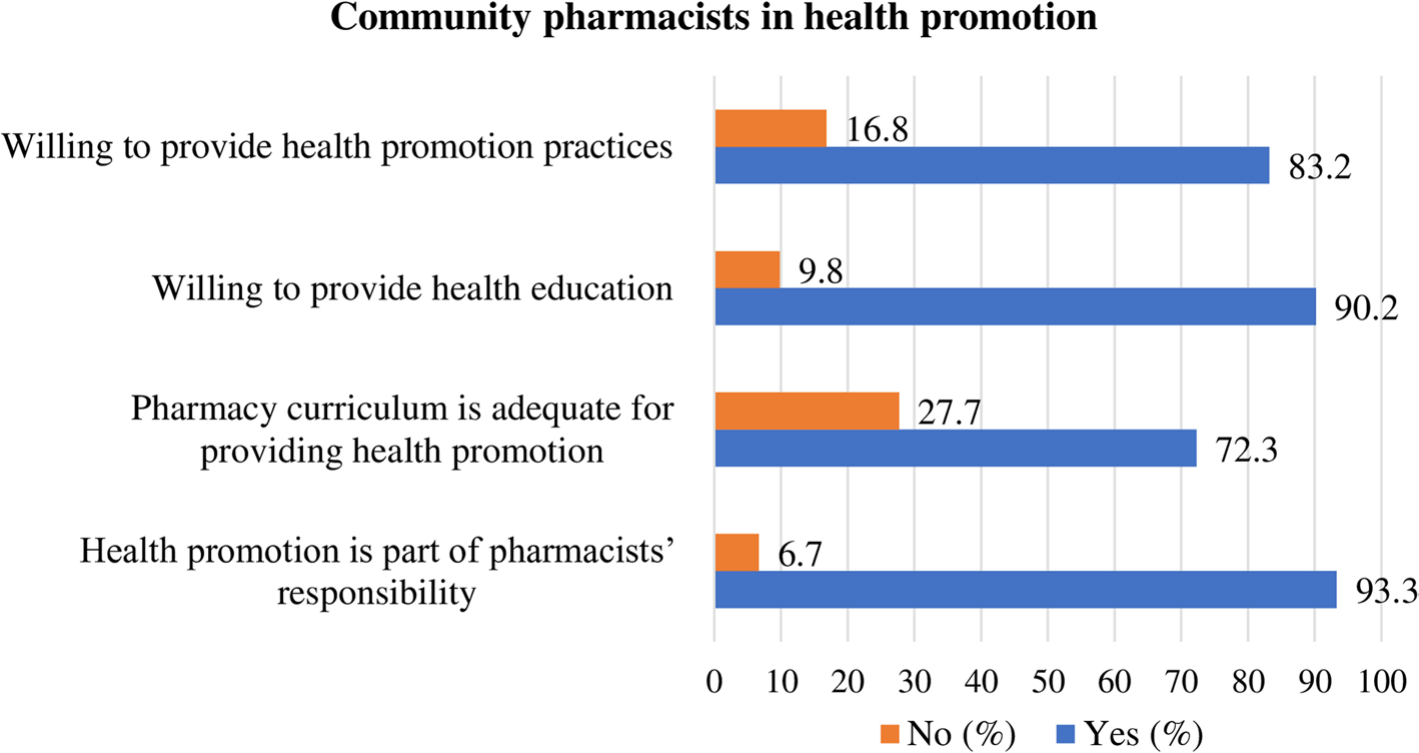
Community pharmacists’ willingness to health promotion practices

Most of the participants responded that health promotion is part of pharmacists’ responsibility (93.3%), and they were willing to provide health promotion and education for patients with NCDs (> 83%). Additionally, around three-fourths (72.3%) also responded that the pharmacy curriculum was adequate for providing health promotion (Fig. 1).

The overall health promotion involvement score of the pharmacists in the prevention and management of the NCDs was 3.82(± 0.60) out of 5. Most of the community pharmacists responded that they were involved or very involved in most of the items assessing the health promotion involvement of pharmacists in the prevention and management of NCDs. More than three-fourths (75.8%) of pharmacists were involved or very involved in promoting service of advising patients on a healthy weight reduction by a non-weight-bearing diet, with an involvement score of 3.85, which is higher than the overall average score. Additionally, most of the community pharmacists were involved or very involved in advising patients on promoting physical activities, alcohol consumption restrictions, smoking cessation, and salt restriction, with an involvement score of greater than four of five points. In contrast, the proportion of pharmacists who were involved in promoting the consumption of cholesterol-lowering diets, giving advice on increased consumption of soluble fibers, screening and measuring blood pressure, weight, and glucose levels, promoting cautions about over-the-counter drugs or herbal products, and monitoring the patients’ response to the treatment was limited, with a mean involvement score that was far below the average mean score (Table 2).

**Table 2:** Community pharmacists’ involvement in health promotion practices in the prevention and management of chronic diseases

Items of health promotion activities	Level of pharmacists’ response on health promotion activities (*n*, %)					Mean score (± SD), out of 5 points
	**Not involved**	**Little involved**	**Uncertain**	**Involved**	**Very involved**	
Promotion of weight reduction by low calorie and non-weight bearing diets	4 (1.4)	40 (14.0)	25 (8.8)	141 (49.5)	75 (26.3)	3.85(± 1.01)
Promotion on physical activity	4 (1.4)	32 (11.2)	17 (6.0)	134 (47.0)	98 (34.4)	4.02(± 0.99)
Promotion on alcohol consumption restriction	5 (1.8)	23 (8.1)	22 (7.7)	123 (43.2)	112 (39.3)	4.10(± 0.97)
Promotion on smoking cessation	7 (2.5)	19 (6.7)	20 (7.0)	125 (43.9)	114 (40.0)	4.12(± 0.97)
Promotion salt restriction	4 (1.4)	21 (7.4)	20 (7.0)	117 (41.1)	123 (43.2)	4.17(± 0.95)
Promotion on consumption of cholesterol-lowering diets	4 (1.4)	38 (13.3)	38 (13.3)	159 (55.8)	46 (16.1)	3.72(± 0.94)
Promotion on consumption of vegetables	4 (1.4)	46 (16.1)	36 (12.6)	131 (46.0)	68 (23.9)	3.75(± 1.04)
Advice on increasing the consumption of soluble fiber	6 (2.1)	46 (16.1)	53 (18.6)	139 (48.8)	41 (14.4)	3.57(± 0.99)
Counsel on cautions of over-the-counter drugs or herbal products	9 (3.2)	70 (24.6)	36 (12.6)	125 (43.9)	45 (15.8)	3.45(± 1.12)
Advice on routine weight, blood pressure and blood glucose monitoring and maintaining the target goals	3 (1.1)	44 (15.4)	15 (5.3)	130 (45.6)	93 (32.6)	3.93(± 1.04)
Involving in screening and measurement of blood pressure, weight and glucose level	9 (3.2)	67 (23.5)	27 (9.5)	110 (38.6)	72 (25.3)	3.59(± 1.19)
Advice on prescription treatment of chronic diseases	1 (0.4)	43 (15.1)	27 (9.5)	151 (53.0)	63 (22.1)	3.81(± 0.96)
Encourage patients’ adherence with treatment	1 (0.4)	27 (9.5)	25 (8.8)	149 (52.3)	83 (29.1)	4.00(± 0.89)
Involving in monitor patients’ treatment response	6 (2.1)	66 (23.2)	47 (16.5)	127 (44.6)	39 (13.7	3.45(± 1.06)
**Community pharmacists’ Overall health promotion involvement score**						**3.82(± 0.60)**

Not involved = 1; little involved = 2; uncertain = 3; involved = 4; very involved = 5

### Pharmacists’ involvement difference in the health promotion activities

An independent sample t-test and one-way ANOVA were employed to compare the mean score difference among participants regarding the overall involvement in health promotion services towards the prevention and management of NCDs. Pharmacists who had a graduating degree and above (Mn = 4.18) were found to have significantly (*p* < 0.001) higher involvement mean scores than druggists (Mn = 3.55). Regarding working hours per day, pharmacists who worked for more than eight hours per day had a higher involvement score (Mn = 3.90) than those who worked less than or equal to eight hours (Mn = 3.72); *p* = 0.012. A one-way ANOVA test also showed that there was a significant difference in the mean involvement score of pharmacists regarding the number of clients served per day (*p* = 0.037). The post hoc test using Tukey’s test disclosed that there was a mean difference between the two pairs: pharmacists who served more than 100 clients/day had a lower involvement score (Mn = 3.50) than those who served 50–100 clients/day (Mn = 3.86); *p* = 0.021) (Table 3).

**Table 3:** Independent samples t-test and one-Way ANOVA analysis to mean score difference among the participants in the provision of health promotion towards NCDs

Variables	Category	Health promotion involvement mean scores		
		**Mean (± SD)**	**t/F**	***P*-value**
Sex	Male	3.80(± 0.48)	0.59^a^	0.556
	Female	3.85(± 0.62)		
Cities where participants involved	Gondar city	3.88(± 0.61)	0.48^b^	0.543
	Bahir Dar	3.80(± 0.59)		
	Debre Tabor	3.78(0 ± 0.60)		
CDRO types where participants worked	Drug store	3.80(± 0.60)	-0.67^a^	0.505
	Pharmacy	3.85(± 0.60)		
Working hours/day	≤ 8	3.72(± 0.62)	-2.52^a^	**0.012**
	> 8	3.90(± 0.58)		
Employment status	Employee	3.81(± 0.61)	-0.34^a^	0.532
	Owner	3.85(± 0.56)		
Pharmacists’ educational level	Druggist	3.55(± 0.57)	-10.51^a^	**0.000**
	Degree and above	4.18(± 0.46)		
Training on health promotion practice:	Yes	3.85(± 0.61)	0.55^a^	0.584
	No	3.81(± 0.60)		
Work experience in years	< 1	3.73(± 0.67)	1.70^b^	0.184
	1–5	3.89(± 0.55)		
	> 5	3.78(± 0.62)		
Monthly income (ETB)	1500–2999	3.77(± 0.67)	0.86^b^	0.423
	3000–4999	3.82(± 0.60)		
	≥ 5000	3.89(± 0.53)		
Number of clients served/day	< 50	3.83(± 0.59)	2.73^b^	**0.037**
	50–100	3.86(± 0.62)		
	> 100	3.50(± 0.52)		

^a^Denotes independent sample t-test; ^b^indicates one-way ANOVA; bold letter at *p*-value indicates *P* < 0.05

### Pharmacists’ level of involvement in the health promotion activities and its determinants

Overall, most pharmacists (58.9%) had a high level of health promotion involvement in the prevention and management of NCDs. This study assessed the potential predictor variables linked to the health promotion activities of community pharmacists in the prevention and management of NCDs. The finding showed that pharmacists who had a bachelor’s degree and above in pharmacy were less likely to have low involvement compared with those who were druggists [AOR = 0.027, 95% CI (0.011–0.63); *p* < 0.001]. Similarly, community pharmacists who served fewer than 50 clients per day were found to be less likely to have low levels of health promotion activities compared with those who served more than 100 clients per day [AOR = 0.194, 95% CI (0.040–944); *p* = 0.042]. In contrast, pharmacists who worked for less than 8 h per day in CDROs were found to be more likely to have low involvement in health promotion services than pharmacists who worked for more than 8 h per day [AOR = 3.645, 95% CI (1.775–7.487); *p* = 0.005] (Table 4).

**Table 4:** Association of pharmacists’ level of involvement towards health promotion activities and predictor variables

Variables		Level of involvement		95% CI		*P*-value
		**Low**	**High**	**COR**	**AOR**	
Sex	Male	70	80	1.638(1.016–2.642)	1.103(0.552–2.207)	0.781
	Female	47	88	1	1	
Educational level	Degree and above	8	116	0.033(0.015–0.072)	0.027(0.011–0.63)	0.000*
	Druggist	109	52	1	1	
Employment status	Employee	95	132	1.178(0.651–2.129)	1.140(0.208–6.241)	0.880
	Owner	22	36	1	1	
Work experience in years	< 1	27	26	1.342(0.683–2.638)	0.887(0.332–2.368)	0.600
	1–5	49	89	0.712(0.416–1.217)	0.685(0.317–1.481)	
	> 5	41	53	1	1	
Monthly salary (ETB)	1500–2999	37	49	1.365(0.719–2.593)	2.447(0.452–13.244)	0.251
	3000–4999	54	72	1.356(0.748–2.458)	1.283(0.241–6.847)	
	≥ 5000	26	47	1	1	
CDRO types where pharmacists worked	Drug store	50	78	0.861(0.535–1.385)	0.611(0.296–1.262)	0.183
	Pharmacy	67	90	1	1	
Number of clients served/day	< 50	62	97	0.349(0.123–0.991)	0.194(0.040–0.944)	0.042*
	50–100	44	65	0.369(0.127–1.072)	0.236(0.049–1.147)	
	> 100	11	6	1	1	
Working hours/day	≤ 8	63	57	2.272(1.400–3.686)	3.645(1.775–7.487)	0.005*
	> 8	54	111	1	1	
Training on health promotion practice	Yes	43	53	1.261(0.767–2.073)	1.201(0.569–2.539)	0.063
	No	74	117	1	1	

*CI* Confidence interval, *COR* Crude odds ratio, *AOR* Adjusted odds ratio

^*^indicates *P* < 0.05

### Barriers to the involvement of pharmacists in health promotion activities

**Fig. 2:**
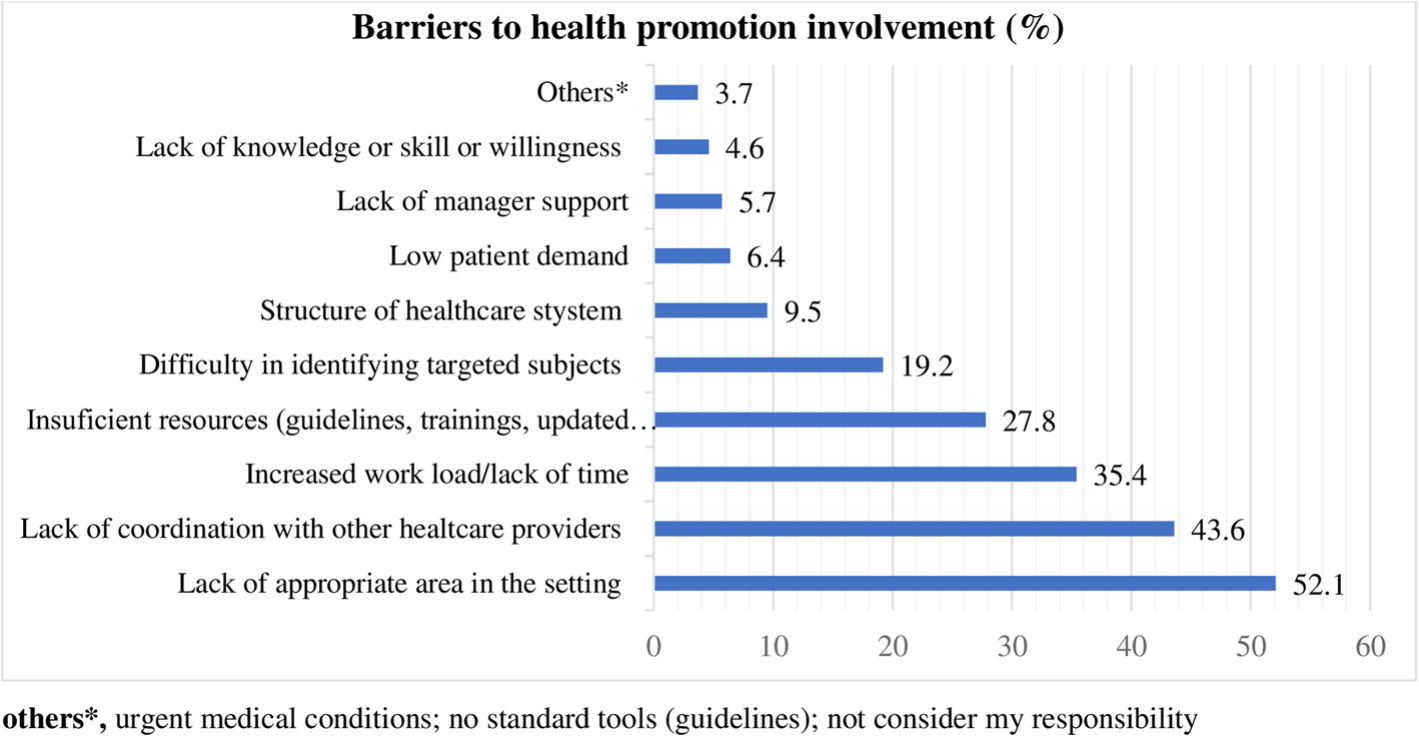
Barriers of community pharmacists in the health promotion practice

Participants responded that the lack of appropriate areas in the CDROs (52.1%), followed by the lack of coordination with other healthcare providers (43.6%), the increase in workload/lack of time (35.4%), and insufficient resources (trainings, guidelines) (27.8%), were the most common reported barriers of community pharmacists in preventing and managing NCDs (Fig. 2).

## Discussion

Although assessing the extent of community pharmacists’ involvement in health promotion activities in the prevention and management of NCDs is critical in tackling the increasing prevalence of NCDs and its associated burdens, comprehensive evidence is lacking in Ethiopia. Because community pharmacists have varying public health roles from their primary dispensing service to a more comprehensive involvement in healthcare issues and are recognized throughout the world [37, 38], assessing their level of involvement and barriers in health promotion activities for NCDs would be vital for taking measures. Thus, this community-based multicenter study explored the willingness, involvement, and barriers of community pharmacists in health promotion activities for the prevention and management of NCDs at CDROs in selected cities in Northwest Ethiopia. Consequently, this study showed that more than half of the community pharmacists had a high level of involvement in health promotion activities. The findings also revealed that differences in educational status and the number of clients per day were associated with the level of involvement. In this study, the lack of appropriate working areas in the CDROs, increased workload, lack of time, insufficient guidance resources and training, and low management support were the most commonly reported barriers.

In this study, more than half of the community pharmacists had a high level of involvement in health promotion for the prevention and management of NCDs. The results highlighted that a most community pharmacists were willing to provide health education and health promotion, and a majority of the participants also agreed that health promotion activities are responsibilities for the pharmacy profession. This indicates that community pharmacists are among the healthcare professionals involved in promoting public health priorities and issues. Currently, community pharmacists provide wide public health services from drug dispensing to medication therapy management, child immunization, health education, screening of diabetes, advice on health risks such as smoking cessation, weight management, blood glucose and blood pressure monitoring, osteoporosis, substance abuse, response to symptoms, and general medication and health information [21, 25, 28, 36]. Consistent with other studies [21, 26, 28, 39–43], in this study, community pharmacists were involved in health promotion activities for the prevention and management of NCDs. Because community pharmacists believe that NCDs are largely associated with obesity, a sedentary lifestyle, and an unhealthy diet, they agreed to give more attention to healthy lifestyle modifications, and they are also involved in lifestyle modification promotion activities such as healthy weight reductions with low-calorie non-weight-bearing diets, promotion of alcohol consumption restrictions, healthy physical activity promotions, and promotion of cigarette smoking cessation, which could decrease the potential risks of NCDs. The result highlighted the fact that most pharmacists have good involvement in NCD health promotion activities for the prevention and management of these disorders. This may implicate those pharmacists have enormously increased their awareness and attitudes about the public’s healthcare priorities and the risks of NCDs. As a result, they are willing to get involved in health promotion activities aimed at preventing and managing NCDs.

This study implies that the majority of community pharmacists were involved with various degrees of involvement, from being involved to being very involved, in most of the important health promotion activities in the prevention and management of NCDs. The reason might be that those pharmacists have been involved in patient education and counseling approaches following the new development of the country’s pharmacy education curriculum, which has undergone a paradigm shift from traditional dispensing-only practices to patient-oriented approaches. Herewith, pharmacists working in CDROs have been graduated after being prepared with various clinical cases and scenarios, along with patients’ approaches and communication skills. In this study, most of the participants also agreed that the curriculum is adequate for providing health care, and they also agreed that health promotion activities are the responsibility of pharmacists. Thereby, this study showed that most of the community pharmacists had a better level of involvement in most of the health promotion activities in the prevention and management of NCDs. Conversely, fewer pharmacists were trained regarding health promotion activities related to the prevention and management strategies of NCDs, which need to be improved to maximize the effectiveness of pharmacists in these services. Therefore, community health authorities, national NCD STEPS authorities, and other stakeholders could have been involved in taking initiatives and endorsements on training and regularly promoting the educational background of the pharmacists. Collaborations between CDROs, health authorities, and relevant educational training centers could be encouraged with the goal of involving community pharmacists in initiatives and workshops promoting health priorities such as NCDs.

In contrast to previous studies [44, 45], this study showed that pharmacists’ involvement in promoting alcohol consumption and smoking cessation was much higher. These findings may indicate that the pharmacists in this study may have observed that an increase in the sedentary lifestyles of the community became their concern and motivated them to become involved in promoting the risks of unhealthy alcohol consumption and smoking habits. Furthermore, in contrast with earlier studies [44, 46], pharmacists’ involvement was low in some health promotion activities, such as promoting the consumption of cholesterol-free and lowering diets, increasing the consumption of soluble fiber, participating in screening and measuring weight, blood pressure, and glucose levels, and monitoring patients’ treatment responses. Lower involvement levels would imply that community pharmacists were not inclusively involved in all basic points concerning the prevention and management of NCDs. This might be because of a gap in skills in some selected areas or the poor commitment of the pharmacists in these areas. But this was correlated with previous studies conducted on the counseling involvement of pharmacists in cardiovascular disorders [25] and metabolic syndrome [26].

The current study also assessed the potential predictor variables linked to the health promotion activities of community pharmacists in the prevention and management of NCDs. Consequently, the finding showed that those pharmacists graduating with a bachelor’s degree and above were more likely to have a high level of involvement in the provision of health promotion services compared with those who were druggists. An independent samples t-test revealed that pharmacists with a bachelor's degree or higher had higher mean health promotion involvement scores than pharmacists with a lower educational level. This study correlated with an earlier study regarding pharmacists’ involvement in preventing cardiovascular diseases [25]. The reason for these disparities could be that pharmacists with a higher level of education may have more up-to-date knowledge and the ability to participate in health promotion activities than pharmacists with a lower level of education. They could also easily access the updated resources and understand how they could be implemented and changed in practice, which helped them become highly involved in health promotion activities for the prevention and management of NCDS. In addition, an earlier survey of bachelor’s degree pharmacy students regarding their health promotion activities while on attachment also revealed that professional training, knowledge, and standard guidelines for the services are important [33]. But in this study, most of the pharmacists had a lower educational background. Therefore, pharmacists would be recommended to upgrade their educational backgrounds to provide better healthcare services in the area of public health priorities such as health promotion aiming to prevent and manage NCDs. Besides their academic education, they must also increase their knowledge, skills, and confidence through training in health promotion services.

Moreover, this study revealed that community pharmacists who served lower numbers of clients per day were found to be more likely to have high involvement in health promotion activities in the prevention and management of NCDs. This could be justified by the fact that those who serve more clients per working day might be busy with other duties and dispensing services. Their counseling services may also go through traditional medicine dispensing rather than adequately engaging in health promotion activities and counseling. The finding may also imply that community pharmacists would serve an optimal number of clients in their working day to provide better counseling, health promotion activities, and healthcare services to patients. Furthermore, pharmacists who worked shorter working hours (8 h/day) in CDROs were found to be less likely to participate in health promotion activities than pharmacists who worked longer working hours (> 8 h/day). This might be because pharmacists working shorter hours may not have better access to clients, and they may use their longer working hours for other duties that are not related to healthcare activities. This finding may indicate those pharmacists could be attached to the optimal number of clients for sufficient hours per working day to increase their healthcare activities in public health priorities like NCDs, which require pharmacists’ involvement.

Although most of the participants had high levels of health promotion involvement in most important health services for the prevention and management of NCDs, many barriers were reported. Community pharmacists reported different types of barriers to being involved in health promotion activities, which need to be addressed to optimize the effectiveness of pharmacists in health promotion activities for the prevention and management of NCDs. Consistent with previous studies [19, 21, 25, 29, 44, 47], the lack of an appropriate working area in the CDROs, increased workload/lack of time, insufficient guidance resources and training, and low management support were the most commonly reported barriers. Additionally, a lack of coordination with other healthcare providers is also an important reported barrier that limits the active involvement of pharmacists in public healthcare priorities like promoting NCDs [19]. Moreover, a study conducted on pharmacy students who were in community pharmacy practice revealed that clients’ lack of time and interest, the absence of a guideline for health promotion services, a lack of training and/or knowledge, and a lack of confidence by pharmacists were the main barriers perceived to hinder the provision of health promotion services. These findings indicate that the barriers are common and similar across different study settings. The findings also suggest that these barriers are multifactorial and related to pharmacy professionals’ knowledge, attitudes, and skills; the structural systems of healthcare; and the clients themselves. Most of the reported barriers are also preventable and modifiable. Therefore, a system could be designed to minimize the effects of barriers and boost the effectiveness of pharmacists in health promotion services. In addition to formal education they received, pharmacists must improve their knowledge, skills, and confidence through formal and informal life-long training in health promotion services. It can reduce the barriers related to pharmacists’ knowledge and skills, and the educational gaps. In particular, the provision of health promotion services for NCDs is crucial because their burden has been increasing in low- and middle-income countries, including Ethiopia. As a result, minimizing the effect of barriers to health promotion practices is among a multifactorial intervention that could be important to tackle the significant burdens associated with NCDs.

Generally, this study has highlighted the levels and extent of involvement and barriers of community pharmacists in health promotion activities for the prevention and management of NCDs. These roles range from promoting lifestyle modification by maintaining a healthy weight with low-calorie diets, promoting physical activities, alcohol consumption restrictions, salt restrictions, and cigarette smoking cessation, to screening and monitoring of weight, blood pressure, glucose levels, and treatment responses, and promoting medication adherence. Therefore, this study may indicate that the rapid rise in the prevalence and burden of NCDs in developing countries like Ethiopia is an urgent call for multisectoral and multidirectional prompt prevention to minimize associated burdens. In fact, promoting healthy behavior among the public is a key population strategy for reducing the burden of NCDs, and this may also be the driving point for community pharmacists to deliver NCD prevention and management.

### Study limitations and strengths

The current study has some limitations. Initially, the findings from this study may not be generalized to all community pharmacists in the country, particularly in rural settings. Data collection may be influenced by participants' honesty and faith in the outcome, resulting in an overestimation or underestimation of current practices and community pharmacists' involvement in NCD health promotion activities. Therefore, the findings of this study should be interpreted with caution. Despite this limitation, this study assessed the extent and level of involvement of community pharmacists in health promotion services in the prevention and management of NCDs at CDROs in selected cities of Northwest Ethiopia, where there is a need for evidence in the area. We hope this study may add a body of knowledge to the existing literature gap in the area, and we believe it will inform policymakers to integrate CDROs with health promotion practices and nationwide efforts to tackle the increasing prevalence of NCDs and their associated burdens in the country. Finally, we recommend that future research investigate the attitudes and beliefs of pharmacists regarding their involvement in the provision of health promotion for noncommunicable diseases in the study settings, which according to existing research, has proven to be very significant.

## Conclusion

Community pharmacists provided NCD health promotion services, and most of them had a high level of involvement in prevention and management strategies. The level of involvement of pharmacists was associated with their level of educational background, the number of clients they provided service to, and their working hours per day. Lack of appropriate counseling areas in the CDROs and lack of coordination with other healthcare providers are the most reported barriers to health promotion involvement that need to be addressed. Pharmacists would be recommended to take training and promote their educational background to a higher level, and coordination with other healthcare providers would be recommended.

## Supplementary Information

**Additional file 1: Supplementary file.** Data collection instrument.

## Data Availability

The datasets generated and/or analyzed during the current study are not publicly available to protect from unnecessary abuse of full data of the participants, but are available from the corresponding author on reasonable request.

## Abbreviations

CDROs: Community Drug Retail Outlets
NCDs: Noncommunicable diseases
