# Supplementary material for: Pharmacists’ involvements and barriers in the provision of health promotion services towards noncommunicable diseases: Community-based cross-sectional study in Northwest Ethiopia
Source: Arch Public Health. 2023 Feb 25;81:31. doi: 10.1186/s13690-023-01038-x (PMC9968412; doi:10.1186/s13690-023-01038-x)
Supplement: Supplementary file 1 — Additional file 1: Supplementary file. Data collection instrument. [file 13690_2023_1038_MOESM1_ESM.docx]

**Supporting information**

Informed consent forms and data collection instruments used for assessing pharmacists’ roles and barriers in health promotion services for the prevention and management of noncommunicable diseases at CDROs in Northwest Ethiopia.

**Informed Consent form**

Dear participant,

We would like to kindly request your consent to participate in the study. The aim of this study is to assess pharmacists’ levels of involvement and barriers in the provision of health promotion towards noncommunicable diseases at CDROs in Northwest Ethiopia. This is a cross-sectional study; the questioner comprises questions regarding your socio-demographic information, willingness and level of involvement in the provision of health promotion services towards prevention and management of noncommunicable diseases, and barriers related to the provision of health promotion. The survey will be conducted among licensed pharmacy professionals who have been working for at least three months. This questionnaire will take you no more than 10–15 minutes, and all the information we obtain will remain strictly confidential, and your answer and name will never be revealed. We assure you that it is totally voluntary, and you are free to refuse or withdraw at any point in the study.

Do you agree to participate in this study? 1. Yes ----                   2. No ----

**Data collection instruments**

Part I: Socio-demographic characteristics

| Sex | 1. Male 2. Female |
| --- | --- |
| Age (in years) | ----------- |
| Cities where you are working | ---- |
| Highest educational qualification | 1. Druggist 2. Bachelor degree and above |
| Work experience | 1. < 1year 2. 1-5 Years 3. >5 years |
| Employment status | 1. Employee  2. Owner |
| Monthly income (birr) | 1. ≤1499 2. 1500-2999 3. >3000- 4999 5. ≥ 5000 |
| Type of Drug retail outlets (CDROs) participants involved | 1. Drug store 2. Pharmacy |
| Average number of clients served per day | 1. <50 2. 50-100 3. >100 |
| Average working hours per day | 1. ≤_8 2. > 8 |
| On work training in health promotion practice | 1. Yes 2. No |

II. Willingness of the pharmacists in the health promotion activities

| Items | Responses | |
| --- | --- | --- |
|  | Yes | No |
| Willing to provide health promotion practices |  |  |
| Willing to provide health education |  |  |
| Pharmacy curriculum is adequate for providing health promotion |  |  |
| Health promotion is part of pharmacists’ responsibility |  |  |

Part III: Involvement of pharmacists in provision of health promotion services for the prevention and management of noncommunicable diseases (**Not involved=1, little involved=2, uncertain=3, involved=4, very involved=5**)

| Items | Not  involved | Little involved | Uncertain | Involved | Very involved |
| --- | --- | --- | --- | --- | --- |
| 1. Promotion on weight reduction by low calorie and non-weight bearing diet |  |  |  |  |  |
| 1. Promotion on physical activity |  |  |  |  |  |
| 1. Promotion on alcohol consumption restriction |  |  |  |  |  |
| 1. Promotion on smoking cessation |  |  |  |  |  |
| 1. Promotion on salt restriction |  |  |  |  |  |
| 1. Promotion on consumption of cholesterol-lowering diets |  |  |  |  |  |
| 1. Promotion on consumption of vegetables |  |  |  |  |  |
| 1. Advice on increase consumption of soluble fiber |  |  |  |  |  |
| 1. Counsel on cautions of over-the-counter drugs or herbal products |  |  |  |  |  |
| 1. Advice on routine weight, blood pressure and blood glucose monitoring and maintaining the target goals |  |  |  |  |  |
| 1. Involving in screening and measurement of blood pressure, weight and glucose level |  |  |  |  |  |
| 1. Advice on prescription treatment of chronic diseases |  |  |  |  |  |
| 1. Encourage patients’ adherence with treatment |  |  |  |  |  |
| 1. Involving in monitor patients’ treatment response |  |  |  |  |  |

Part IV: Possible barriers of community pharmacists in provision of health promotion in the prevention and management of noncommunicable diseases (**can choose more than one options**)

| Barriers to involvement (**you can choose more than one possible barrier**) |
| --- |
| 1. Lack of time or increase workload |
| 1. Difficulty in identifying targeted subjects |
| 1. Lack of pharmacists’ knowledge, skills or willingness |
| 1. Lack of appropriate area |
| 1. Lack of manager support |
| 1. Lack of coordination with other healthcare providers |
| 1. Insufficient resources (guidelines, manuals, trainings,) |
| 1. Structure of healthcare system |
| 1. Others (**if possible**) |
|  |
|  |
|  |

**Thank you for participation!**
